# Cellular and molecular determinants mediating the dysregulated germinal center immune dynamics in systemic lupus erythematosus

**DOI:** 10.3389/fimmu.2025.1530327

**Published:** 2025-02-13

**Authors:** Spiros Georgakis, Kalliopi Ioannidou, Bernat Bramon Mora, Michail Orfanakis, Cloe Brenna, Yannick D. Muller, Perla M. Del Rio Estrada, Ashish A. Sharma, Giuseppe Pantaleo, Laurence de Leval, Denis Comte, Raphael Gottardo, Constantinos Petrovas

**Affiliations:** ^1^ Department of Laboratory Medicine and Pathology, Institute of Pathology, Lausanne University Hospital and Lausanne University, Lausanne, Switzerland; ^2^ Biomedical Data Science Center, Lausanne University Hospital and Lausanne University, Lausanne, Switzerland; ^3^ Service of Immunology and Allergy, Department of Medicine, Lausanne University Hospital and University of Lausanne, Lausanne, Switzerland; ^4^ Pathology Advanced Translational Research Unit, Department of Pathology, Emory University School of Medicine, Atlanta, GA, United States; ^5^ Centro de Investigación en Enfermedades Infecciosas, Instituto Nacional de Enfermedades Respiratorias “Ismael Cosío Villegas”, Mexico City, Mexico; ^6^ Service of Internal Medicine, Department of Medicine, Lausanne University Hospital and University of Lausanne, Lausanne, Switzerland; ^7^ Swiss Institute for Bioinformatics, Lausanne, Switzerland

**Keywords:** T follicular helper cells (T_FH_), type I IFN, age-associated B cells, germinal center response, IL-4, systemic lupus erythematosus (SLE)

## Abstract

**Introduction:**

Systemic lupus erythematosus (SLE) is characterized by dysregulated humoral immunity, leading to the generation of autoreactive B cells that can differentiate both within and outside of lymph node (LN) follicles.

**Methods:**

Here, we employed spatial transcriptomics and multiplex imaging to investigate the follicular immune landscaping and the *in situ* transcriptomic profile in LNs from SLE individuals.

**Results:**

Our spatial transcriptomic analysis revealed robust type I IFN and plasma cell signatures in SLE compared to reactive, control follicles. Cell deconvolution revealed that follicular T cell subsets are mainly affected by the type I IFN fingerprint of SLE follicles. Dysregulation of T_FH_ differentiation was documented by i) the significant reduction of Bcl6^hi^ T_FH_ cells, ii) the reduced cell density of potential IL-4 producing T_FH_ cell subsets associated with the impaired transcriptomic signature of follicular IL-4 signaling and iii) the loss of their correlation with GC-B cells. This profile was accompanied by a marked reduction of Bcl6^hi^ B cells and an enrichment of extrafollicular CD19^hi^CD11c^hi^Tbet^hi^, age-associated B cells (ABCs), known for their autoreactive potential. The increased prevalence of follicular IL-21^hi^ cells further reveals a hyperactive microenvironment in SLE compared to control.

**Discussion:**

Taken together, our findings highlight the altered immunological landscape of SLE follicles, likely fueled by potent inflammatory signals such as sustained type I IFN and/or IL-21 signaling. Our work provides novel insights into the spatial molecular and cellular signatures of SLE follicular B and T_FH_ cell dynamics, and points to druggable targets to restore immune tolerance and enhance vaccine responses in SLE patients.

## Introduction

SLE represents the prototypic systemic autoimmune disease affecting predominantly women of childbearing age. Genetic and environmental factors lead to systemic and extensive dysregulation of both innate and adaptive arms of the immune system resulting in aberrant autoreactive responses. Two of the main interrelated hallmarks of SLE are the presence of autoantibodies (auto-Abs) in the patients’ sera, and the profound expression of type I IFN stimulated genes (ISGs) in blood and non-blood inflamed tissues (1–3). Autoantibodies bind to apoptotic or necrotic material released from dying cells and form immune complexes (ICs) that can activate IFN-producing cells, like plasmacytoid dendritic cells (pDCs), to secrete copious amounts of type I IFNs such as IFNα2 (4, 5). Subsequently, type I IFNs can potentiate autoreactive immune responses creating a deleterious feedback loop. Type I IFNs exert immunomodulatory effects on both the blood and secondary lymphoid tissues of SLE humoral responses at multiple levels including the enhancement of i) plasma cell differentiation and/or survival (6–8), ii) generation of non-canonical autoantibody secreting B cell subsets like age-associated-B cells (ABCs) (1–3), iii) TLR-fueled B cell-responsiveness to autoantigens (9) and by modulating T cell -mediated B cell maturation (10). The highly inflammatory microenvironment in SLE can affect both follicular (F) and extrafollicular (EF) responses taking place in lymph nodes (LNs) (11).

Previous findings have shown that most IgG autoantibodies detected in SLE patients’ sera are somatically mutated supporting the involvement of germinal centers (GCs) in SLE humoral responses while a fraction of antibody-secreting cell (ASCs) clones contained unmutated autoantibodies, a sign of GC-independent B cell differentiation (12). Notably, extrafollicular GC-independent responses are loosely regulated and can lead to the generation of autoreactive B cell clones (13). On the other hand, it is well-established that efficient GC-responses require T follicular cells (T_FH_) cells (14). These cells are mainly found in the follicles of secondary lymphoid organs and are characterized by their capacity to mediate B-cell clonal expansion, antigen-based affinity maturation and plasma cell differentiation in an IL-21- and IL-4- dependent manner (15). T_FH_ cells can be phenotypically distinguished by their high expression of Bcl6, PD-1 and CXCR-5 (16, 17). Even if they represent a small fraction of T cells, T_FH_ cells exhibit high phenotypic and functional heterogeneity (15, 18) as well as adaptiveness to tissue microenvironment (19). T_FH_ cell subsets are endowed with distinct phenotypes, GC-localization patterns, and cytokine-secreting potential (18). Several studies provided evidence that deregulated somatic hypermutation taking place in GCs can give rise to autoantibody-secreting B cells highlighting the importance of proper T_FH_-mediated reactions (20–22). Previous findings also revealed that circulating T_FH_-like cells (CD4^+^PD1^+^CXCR5^+^) are up-regulated in SLE patients’ peripheral blood and correlated with SLE disease activity score (SLEDAI) (23). Whether circulating T_FH_-like cells represent blood counterparts of bona fide GC T_FH_ cells or a memory subset (memory T_FH_) that leave GCs due to incomplete interaction with B cells is not well understood (24). Additionally, deregulated T_FH_ cell responses in SLE might be the cause of impaired vaccine responses as previously reported (25, 26). Due to the difficulty in obtaining relevant human material, the *in-situ* investigation of the immune landscape of secondary lymphoid organs in SLE is still an understudied area of research.

Herein, by using a combination of spatial transcriptomics and multiplex imaging analysis of SLE with an active disease compared to reactive, non-autoimmune control LNs, we provide evidence for a profound type I IFN signature associated with an altered immune landscape characterized by dysregulated T_FH_ and B cell dynamics, which may contribute to the generation of autoreactive B cell subsets in SLE LNs. Further understanding of deregulated GC responses is of great interest to ameliorate SLE symptoms and improve vaccine responses in SLE patients.

## Materials and methods

### Human subjects

The tissue samples used in this study were obtained from the archives of the Institute of Pathology of Lausanne University Hospital, Switzerland (**Supplementary Table S1** **,** **Supplementary Figure S1A**). LN cells were obtained from the Centro de Investigacion en Enfermedades Infecciosas (CIENI), Instituto Nacional de Enfermedades Respiratorias (INER) in Mexico City, Mexico. All procedures were in accordance with the Declaration of Helsinki and approved by i) the Canton de Vaud-CER-VD, Switzerland for control LN tissues (#2021-01161), ii) the local research consent authorities for LN analysis and the reuse of clinical data from the five SLE patients at CHUV, with oral consent provided by the patients after a thorough explanation of the study by the investigators, and iii) the Research Committee and the Ethics in Research Committee of the National Institute of Respiratory Diseases “Ismael Cosío Villegas,” Mexico City as part of the ‘C71-18’ protocol.

### Tissue processing

Fresh tissues samples were promptly fixed overnight in formalin, following biopsy, and processed into paraffin embedded (FFPE) blocks using standard procedures. All subsequent tissue processing was carried out in our Institute. The blocks were sequentially cut into 4 μm sections and prepared on Superfrost glass slides (Thermo Scientific, Waltham, MA, USA, Ref. J1800AMNZ), dried overnight at 37°C and stored at 4°C. Before staining, the slides were heated on a metal hotplate (Stretching Table, Medite, Burgdorf, OTS 40.2025, Ref. 9064740715) at 65°C for 20 min. This melting step ensures the proper adherence, deparaffinization and optimal epitope exposure of the tissue section.

### Tissue spatial transcriptomic analysis

Transcriptomic profiling was performed using the commercially available platform GeoMx Digital Spatial Profiling (Nanostring) according to the manufacturer’s instructions. 4 µm FFPE tissue sections from SLE (N=4) and control (N=3) LN were used. Follicular Regions of Interest (ROIs, n=9-12) were identified based on the CD3, CD20, PD-1 *in situ* staining pattern (active follicles/characterized as CD20-dense areas populated by CD3^+^PD1^+^ cells were selected for analysis) before the probe-hybridization step. The average ROI area was 126336.4 µm² (± 59758.8728, SD) for control and 157725.2281 µm² (± 78722.80994, SD) for SLE ROIs.

For data Processing and quality control (QC), we followed standard GeoMx processing workflows (https://doi.org/10.18129/B9.bioc.GeoMxWorkflows.). The processing of DCC files and quality checks at various levels (segment, probe, and gene) were carried out with the help of the ‘GeoMxWorkflows’(https://doi.org/10.18129/B9.bioc.GeoMxWorkflows.),’GeomxTools’(https://doi.org/10.18129/B9.bioc.GeoMxWorkflows.) and ‘NanoStringNCTools’ (https://doi.org/10.18129/B9.bioc.NanoStringNCTools.). packages in R (version 4.3.2). First, we adjusted all zero expression counts to one to facilitate downstream data transformation. Next, we applied several quality control cut-offs recommended by NanoString, including: a minimum of 1000 reads, 80% trimming, stitching, and alignment, 50% sequencing saturation, a minimum negative control counts of 1, a maximum of 1000 reads observed in NTC wells, and a minimum area of 1000. In addition to the segment quality control, probes with an average expression count across segments below 10% of the average for other probes targeting the same gene were excluded. Likewise, we also removed probes deemed outliers in over 20% of segments, using Grubb’s test (https://doi.org/10.18129/B9.bioc.GeoMxWorkflows.) as the outlier detection method. Finally, we excluded segments where less than 5% of the genes in the panel were detected above the quantification limit (LOQ, calculated as two standard deviations beyond the mean), and genes falling below the LOQ in at least 10% of the segments.

To perform batch correction, we first normalized the raw data using the Trimmed Mean of M-values (TMM) method with the R package “standR” 15, which adjusts for differences in library sizes and composition between RNA-seq samples. We then applied the RUV-4 correction from the same package 16, which removes unwanted variations by identifying negative control genes and calculating scaling factors for batch correction. In our analysis, we identified several negative control genes and set the number of scaling factors to 2.

To correct for batch effects, we first normalized the dataset using the Trimmed Mean of M-values (TMM) method, implemented via the ‘standR’ package in R (https://doi.org/10.18129/B9.bioc.standR). This method adjusts for discrepancies in library size and composition between RNA-seq samples. Following this, we applied the RUV-4 correction (27), which uses negative control genes to calculate scaling factors and remove unwanted variability due to batch effects. In this study, we identified 300 negative control genes and set the number of scaling factor to 3 for the RUV-4 correction.

For the differential gene expression analysis, we employed the limma-voom workflow (28, 29). That is, we built a linear model using a design matrix that accounted for the treatment variable and RUV-4 correction scaling factors as covariates. We then identified differentially expressed genes between treatments based on an adjusted p-value cut-off of less than 0.05. Finally, we performed gene set enrichment analysis (GSEA) on the differentially expressed genes to identify biological pathways of interest (30). We applied the ‘fry’ method from the R package ‘limma’ (28) and used canonical pathway gene sets from the Reactome (https://doi.org/10.18129/B9.bioc.msigdb.). The results were then analyzed and visualized using the R package ‘vissE’ (https://doi.org/10.18129/B9.bioc.vissE).

### Cell deconvolution

To perform cell deconvolution on the GeoMx data, we followed the ‘SpatialDecon’ R package pipeline (31). We used lymph node single-cell RNA-seq data from HIV patients as our reference dataset. We focused on cells classified as T cells (CD4, CD8, T_FH_), B cells and dendritic cells. Likewise, we only considered genes expressed in immune and stromal cells (genes used in ‘safeTME’ cell profile matrix from ‘SpatialDecon’) and some additional immune cell-specific genes if needed. To avoid either different population sizes across or genetic variability within cell types to interfere with the deconvolution methods, we first clustered each cell type into smaller populations with well-defined gene signatures, using a k-means algorithm with a total of 15 centers (32). Then, using these new annotations, we generated the lymph-node specific cell profile matrix with ‘SpatialDecon’, and perform the deconvolution on the GeoMx data (31). To study the effects of deconvolution-derived cell proportions on gene expression, we designed several modelling strategies to address the relationship between the expression of specific genes and cell populations. Preliminary tests of the association between gene expression and deconvoluted data generated ΄noisy΄ results, mainly because our deconvolution approach yielded unsatisfactory outcomes in terms of successfully distinguishing the relevant T cell subsets. For this purpose, we merged all T cell populations together, considering that our imaging results proved that most T cells in our ROIs (follicles) are T_FH_. We used the R package ‘DESeq2’ (33) to model gene counts with a negative binomial regression as a function of the RUV-4 scaling factors, segment area, treatment, and the percentage for a given cell population. We applied two models: a first model without the treatment effect and a slope for the cell percentage, and a second model with the treatment effect and treatment-specific slopes for the cell percentage. These models allowed us to understand the extent to which the presence of a particular cell population in each segment is related to the expression levels of specific genes.

### LN scRNA analysis

Cells from viremic HIV LNs were used for the generation of a data set to support the GeoMx cell deconvolution analysis. Briefly, FASTQ files were uploaded to Cell Ranger (10X Genomics cloud), and no depth normalization was carried out. The generated filtered count matrix was further analyzed using the Seurat package in R. Doublet cells were removed from the analysis using the DoubletFinder package in R. Cell annotations were performed using SingleR and the reference expression dataset was derived from the Monaco Immune Data atlas from the cell dex R package. Differentiation gene expression was assessed using the MAST R package. Sequencing data were deposited in the GEO database under the accession ID GSE288212.

### Multiplex immunofluorescence imaging

Multiplex antibody staining was performed on the Ventana Discovery Ultra Autostainer (Roche Diagnostics) as previously described (34). Briefly, the procedure is consisted of consecutive rounds of antigen retrieval, antibody blocking steps (using the Opal blocking/antibody diluent solution) for non-specific binding of antibodies, staining with primary antibodies (details on antibodies, clones and panel are listed in **Supplementary Tables S2, S3**), incubation with secondary HRP-labeled antibodies for 16 min, then detection with optimized fluorescent Opal tyramide signal amplification (TSA) dyes (Opal 7-color Automation IHC kit, from Akoya, Ref. NEL821001KT). Repeated antibody denaturation cycles were introduced. Tissue sections stained with Alexa-Conjugated antibodies (panel 5) were incubated with the primary Ab for 90 min and with the secondary Ab (if needed) for 90 min at RT. Just for panel 5, a cycling staining approach was followed. After staining with Alexa-conjugated Abs, the Ab complexes were dissociated using a citric acid-based buffer and a second staining cycle followed with Opal-coupled Abs. The images were aligned using SimpleITK (35) as an Imaris extension (Imaris software version 9.9.0, Bitplane) using a common marker (specifically a nuclear dye). The samples were counterstained with Spectral DAPI from Akoya Biosciences (NEL821001KT) for 4 min or SYTO45 (1/10000 dilution in TBS-T, CatNo10297192, ThermoFischer Scientific) for 35 min, rinsed water with soap and mounted using DAKO mounting medium (Dako/Agilent, Santa Clara, CA, USA, Ref. S302380-2).

### Data acquisition

Multispectral images (MSI) were acquired using the Vectra Polaris imaging system from Akoya or the Leica Stellaris 8 SP8 confocal system. Images (512x512 and 1024x1024 resolution) were acquired using 0.75× optical zoom and a 20× objective (NA) (unless otherwise specified) for all the images used for quantification. Frame averaging or summing was never used while acquiring the images. At least 80% of each section was imaged, to ensure an accurate representation and minimize selection bias. For images acquired with Vectra Polaris dye unmixing was conducted using inForm image analysis software, version 2.4.8 (Akoya Biosciences, Marlborough, MA 01752, USA) and for those acquired with Leica Stellaris 8 we used Leica LAS-AF Channel Dye Separation module which was included in LAS-X (Leica Application Suite X (LAS-X)-4.6.1.27508 software). All the tissues stained for the same panel were imaged using the same platform to be able to process them all together and quantify their cell densities (normalized cell counts per mm^2^) and/or frequencies.

### Quantitative imaging analysis

For images acquired using the Vectra Polaris the Phenochart 1.0.12 software (Akoya Biosciences, Marlborough, MA 01752, USA), a whole-slide contextual viewer with annotation capability was used for navigation around slides and for identification of Regions of Interest (ROIs). MSI were analyzed using the inForm image analysis software, version 2.4.8 from Akoya. Firstly, the images were unmixed and were segmented using CD20, PD-1, KI67 and DAPI as components for training into specific tissue ROIs (GC, non-GC CD20-enriched area, low CD20 area). Individual cells were segmented using the counterstained-based adaptive cell segmentation algorithm, with the help of nuclear (DAPI and BCL-6) and membrane (CD4) markers. Following tissue and cell segmentation, the phenotyping configuration was used, by assigning around 100 cells to the positive phenotype for each marker, while selecting additional 100 cells characterized as “other” for the negative phenotype, choosing across several images. The quantification was based on PhenoptrReports from Akoya Biosciences, an automated R-script platform, where separated merged cell segmentation data, retaining the same tissue segmentation, were created for each phenotyped marker and were consolidated afterwards. The different combinations of phenotyped populations were defined and the analysis was run creating reports, which contained the number of analyzed fields (slide summary), cell counts, cell percentages, and cell densities.

Quantitative data was generated from images captured with Leica Stellaris using Histo-cytometry analysis (given the low abundance of PD1^hi^CD57^hi^ GATA3^hi^ cells, images from panel 3 were analyzed using both approaches as a cross validation step), as previously reported (34, 36). In brief, the Surface Creation module of Imaris software (version 9.9.0 Bitplane) was used to generate 3-dimensional segmented surfaces (based on the nuclear signal) of unmixed images. Data generated from Histo-cytometry, such as average voxel intensities for all channels, in addition to the volume and sphericity of the 3-dimensional surfaces, were exported in Microsoft Excel format. The files were converted to comma separated value (.CVS) files, and the data were imported into FlowJo (version 10) to be further analyzed and quantitated. Well-defined areas devoid of background staining were included in the analysis, and the data were quantified either as relative frequencies or as cell counts normalized to the imaged follicular area. The area and other morphological characteristics (circularity, solidity) of individual follicles were calculated using FIJI software (37). Optimal z-stack settings were applied in all collected images. Maximum Intensity Projections (MIPs) are presented throughout the manuscript.

### Data analysis–neighboring analysis

The distance between relevant cell subsets (CD20^hi/dim^Ki67^hi^, PD1^hi^CD57^hi^, PD1^hi^GATA3^hi^) was calculated with Python 3.10.9 using the SciPy library (38). X and Y coordinates were used to create the matrix interaction for each cell phenotype, and the median distance was also extracted. Additionally, to calculate the probability of observing different patterns of cellular distribution across ROIs and individuals, we studied the curves generated from the Ripley’s G function and the theoretical Poisson curve using pointpats 2.3.0 (https://doi.org/10.5281/zenodo.7706219). The area between the empirical and theoretical Poisson curve was extracted using the NumPy library (39). ROIs harboring at least 20 positive cells for each cell subset under investigation were analyzed.

### Statistical analysis

The Mann-Whitney test and simple linear regression analysis were used to analyze the imaging data. Analyses and graphs were generated using GraphPad Prism 8.3.0 software. Regarding the ROI measurements (Area, Circularity, Solidity), we applied a Mixed Effect Model using a python script comprising both fixed effects corresponding to various follicular areas and random effects originating from individual donors. A p-value < 0.05 was considered statistically significant.

## Results

### SLE follicles are characterized by a profound *in situ* type I IFN and plasma-cell transcriptomic profile

**Figure 1:**
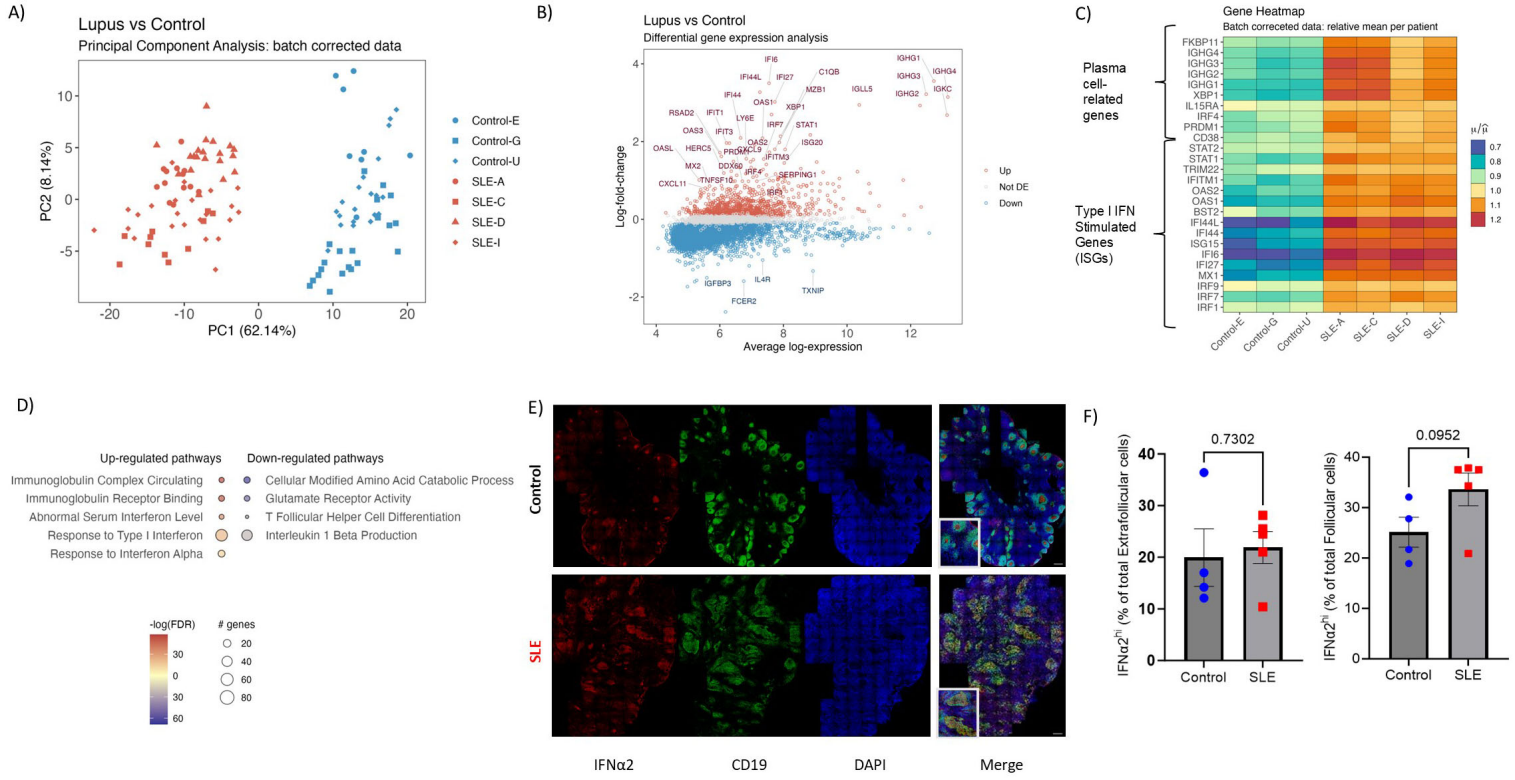
Spatial transcriptomics reveal a potent type I IFN and plasma cell gene signature in SLE follicles. **(A)** The PCA plot shows the distribution of batch corrected spatial transcriptomic data from 111 Regions of Interest (ROIs, secondary follicles) collected from SLE (N=64, 4 donors) and control LNs (N=47, 3 donors). Each point represents an individual ROI, with the color indicating the different cohort (Red=SLE, Blue=Control). **(B)** Volcano plot displaying the differentially expressed genes in SLE and control follicular ROIs. The x-axis represents the average log gene expression between SLE and control follicles, while the y-axis represents the log fold-change in gene expression. Selected genes with significant differential expression (FDR < 0.05) are highlighted: those upregulated in SLE are marked in red and those downregulated in SLE or upregulated in control are marked in blue. Non-significant genes are shown in gray. Selected key genes of interest are labeled. **(C)** Heatmap showing the relative mean expression of type I IFN (lower panel) and plasma cell (upper panel) signature genes. All genes displayed in **(C)** are significantly up- regulated in SLE compared to control ROIs (P value < 0.05). **(D)** Gene ontology pathway analysis performed on differentially expressed genes of SLE and control follicular ROIs. The size of each node represents the number of genes involved in the corresponding GO term, while the color indicates the significance level of the enrichment (-logFDR). **(E)** Representative mIF images of IFNα2 (red), CD19 (green) and DAPI (blue) from SLE and control LNs (scale bar: 500 μm). Zoomed areas are shown in white boxes at the bottom left of the merged image. **(F)** Bar graphs demonstrating the cell frequencies of extrafollicular (left) and follicular (right) IFNα2^hi^ cells in SLE (N = 5) and control LNs (N = 4). Each dot/square represents one donor. The p values were calculated using the Mann–Whitney test. Data represent mean ± SEM.

To investigate the unique characteristics of SLE follicular landscape, we collected LN sections from treatment-naive active SLE patients exhibiting lymph node involvement and from non-autoimmune, cancer- and HIV-free control donors harboring hyperplastic, active follicles (**Supplementary Table S1**) (**Supplementary Figure S1A**). Despite the different etiology of follicular activation/maturation, our selected non-autoimmune tissues represent a strict control group, providing a powerful evaluation for the SLE follicular profiling. To start gaining insights on the complex molecular profile of SLE follicles, we performed spatial transcriptomic analysis using the GeoMX Digital Spatial Profiler platform. Secondary mature follicles were defined as CD20^hi/dim^-dense microanatomical structures populated by CD3^hi^PD1^hi^ (T_FH_) cells (**Supplementary Figure S1B**). Principal component analysis (PCA) of batch-corrected sequencing data revealed that SLE follicles exhibit a transcriptionally distinct profile compared to non-autoimmune reactive controls (**Figure 1A**, **Supplementary Figure S1C**). Volcano analysis showed that several inflammation-related genes (*STAT1*, *CXCL9*, *CXCL11*, *IRF1* etc.) were upregulated in SLE over non-autoimmune control follicles, while IL4-related (*IL4R*, *FCER2*) and cellular oxidation-related (*TXNIP*) genes were upregulated in control follicles (**Figure 1B**). Notably, most of the top upregulated Differentially Expressed Genes (DEGs) in SLE follicular areas were interferon stimulated- (ISGs like *IRF7*, *IFI6*, *IFI44L*, *ISG20* etc) or plasma cell-related (*PRDM1*, *IRF4*, *IGHG1* etc) genes, a profile independent of the gender of SLE individuals. (**Figure 1C**). In line with the DEGs profile, we detected significantly higher expression of type I IFN and immunoglobulin complex pathways in SLE compared to control follicles (**Figure 1D**). Compared to SLE, control follicles were characterized by significant enrichment of T_FH_ cell differentiation and IL1b-related pathways (**Figure 1D**).

To further validate the potent transcriptomic type I IFN signature of SLE follicles a mIF assay, using an antibody against IFN-α2 (panel 4, **Supplementary Tables S2, S3**), was applied (**Figure 1E**). To analyze and quantify IFNα2^hi^ cells in follicles (defined as CD19-dense areas), we employed the histo-cytometry approach (40) (**Supplementary Figure S2A**). Contrary to extrafollicular area, a clear trend (p=0.0952) towards increased levels, both as frequency or normalized counts, of IFN-α2^hi^ cells in SLE compared to control follicles was found (**Figure 1F**, **Supplementary Figure S2B**), further supporting our transcriptomic findings. Altogether, our findings revealed a highly inflammatory follicular microenvironment, dominated by a type I IFN signature, that could affect the development of T_FH_/GC-B cell responses in SLE.

### Type I IFN signature of SLE follicles is mainly assigned to T and dendritic cells

**Figure 2:**
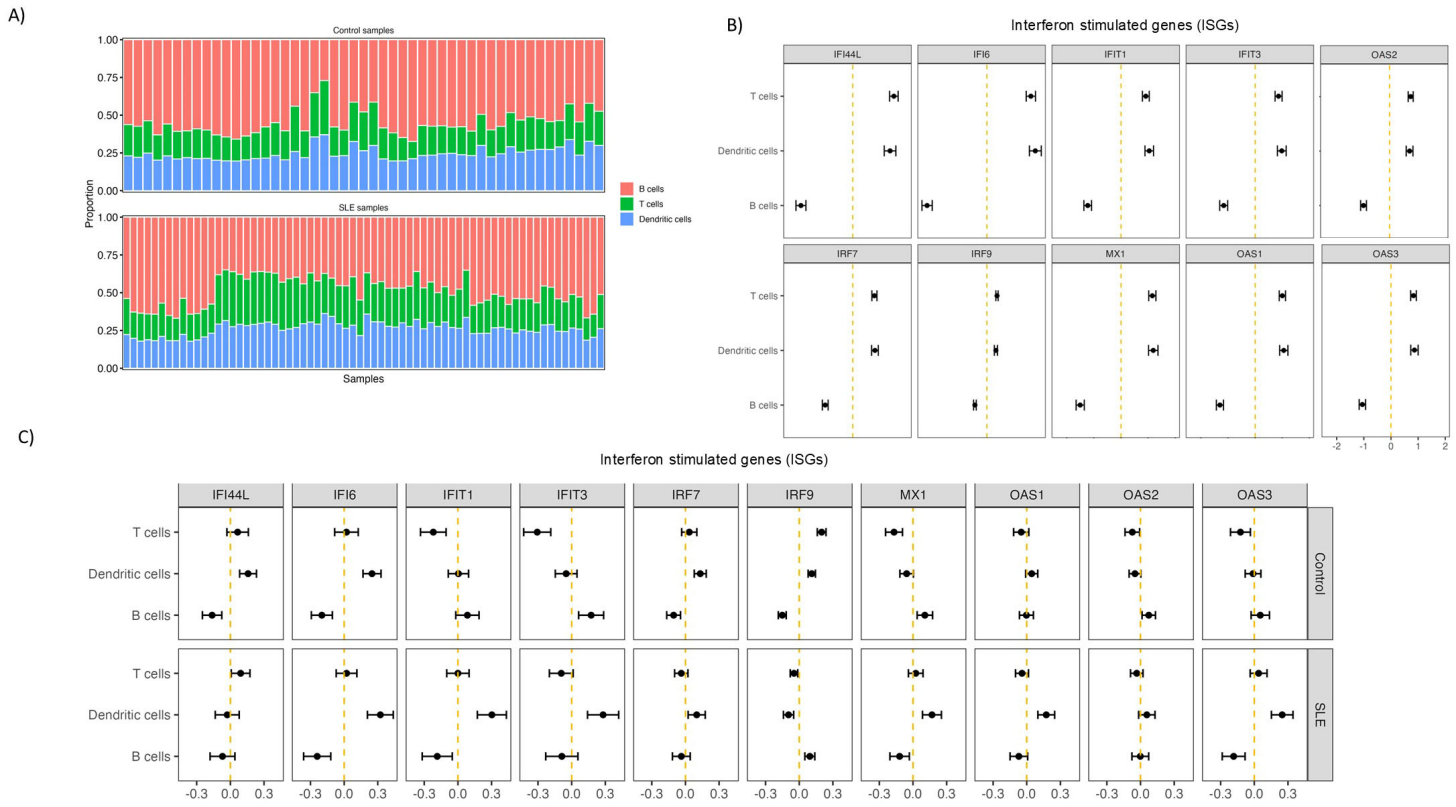
Follicular T cells are more affected by type I IFN signalling than B cells in SLE. **(A)** Bar charts showing deconvoluted GeoMx data indicating the relative abundance of different analyzed cell subsets in control and SLE ROIs. Each column represents a different ROI. Different cell subsets are labelled with different colors. **(B)** Graphs representing the relative correlation of deconvoluted cell subsets proportion (T, B, dendritic cells) from all the ROIs with randomly selected Interferon Stimulated genes. **(C)** Graphs representing the relative correlation of deconvoluted cell subsets proportion (T, B, dendritic cells) from control (upper row) and SLE (lower row) LN ROIs separately with randomly selected Interferon Stimulated genes.

Given the potential expression of IFNAR by several follicular cell types (41), we aimed to investigate the main cellular targets of the observed type I interferon transcriptomic signature in SLE. To this end, we applied a cell deconvolution pipeline using a single-cell dataset from human LNs as a reference. As an internal validation of our pipeline, we found that selected T (e.g. *CD3, TRAC*) and B (e.g. *CD19, AICDA, MS4AI*) cell genes were correctly assigned to the corresponding cell types (**Supplementary Figure S2C**). The approximate calculated relative proportions of T and B cells showed a dominance by B cells (≈50%) with T cells occupying a ≈25% of total deconvoluted cells as expected (**Figure 2A**). We then sought to determine the contribution of T, B and dendritic cell types on interferon stimulated gene (ISG) expression. First, using the full dataset, consisted of both control and SLE ROIs, we observed a higher contribution of T and dendritic cells, compared to B cells, in the observed ISG expression profile (**Figure 2B**). By dividing the dataset into control and SLE ROIs, we found that DCs have a higher contribution to the increased expression of ISGs in the SLE group, a finding consistent with previous single-cell RNA sequencing studies in blood suggesting that innate immune cells have a stronger IFN fingerprint than adaptive immune cells in SLE (42) (**Figure 2C**). Furthermore, for SLE ROIs, a pattern favoring T cell correlation with increased ISG expression compared to B cells was observed for most of the ISGs while B cell correlation was higher only in one case (*IRF9*) (**Figure 2C**). Therefore, our deconvolution approach suggests that follicular T cells are presumably more responsive to type I IFN signaling compared to B cells, despite the expression of IFNAR by both cell types.

### Altered T_FH_ cell differentiation dynamics in SLE

**Figure 3:**
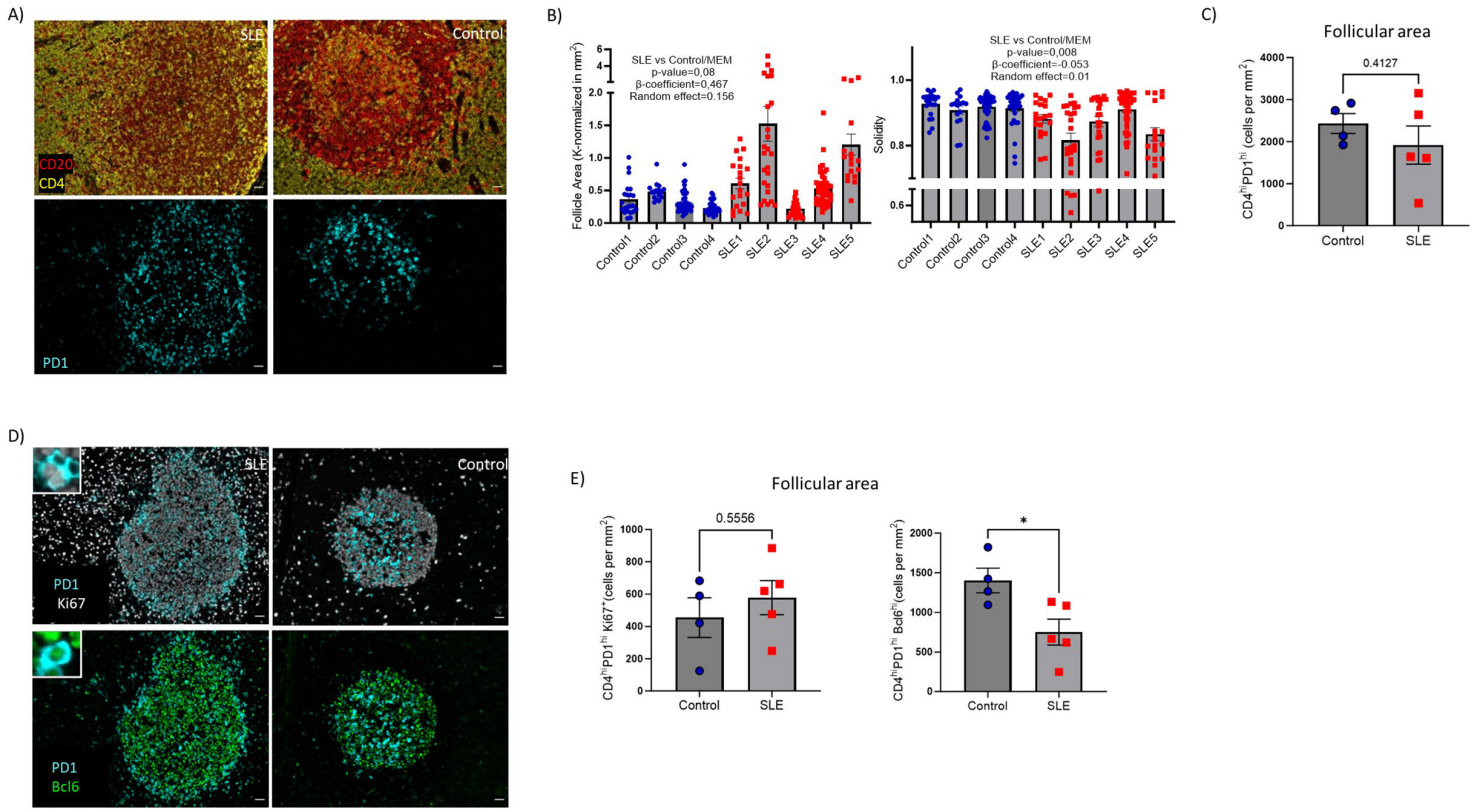
Decreased cell density of Bcl6^hi^ T_FH_ cells in SLE follicles. **(A)** Representative mIF images of CD20 (red), CD4 (yellow) and PD-1 (cyan) from SLE and control LNs (left panel, scale bar:20mm). **(B)** Bar graphs demonstrating the quantification of morphological properties (area, Solidity) of follicles (identified as CD19^hi/dim^ areas. Area and solidity of ROIs were calculated using FIJI. Each dot/square represents a different follicle. The p values were calculated using the mixed effects model (MEM). Data represent mean ± SEM **(C)** Bar graph demonstrating the cell densities of CD4^hi^PD1^hi^ T_FH_ cells in SLE (N = 5) and control follicular areas (N = 4). Each dot/square represents one donor. The p values were calculated using the Mann–Whitney test. Data represent mean ± SEM. **(D)** Representative mIF images of Bcl6 (green), Ki67 (grey) and PD-1 (cyan) from SLE and control LNs (left panel, scale bar:20mm). Zoomed areas are shown in white boxes. **(E)** Bar graphs demonstrating the cell densities of CD4^hi^PD1^hi^Ki67^hi^ (upper) and CD4^hi^PD1^hi^Bcl6^hi^ (lower) T_FH_ cells in SLE (N = 5) and control LNs (N = 4). Each dot/square symbol represents one donor. The p values were calculated using the Mann–Whitney test. Data represent mean ± SEM. *P < 0.05.

The above-mentioned spatial transcriptomic profile of SLE follicles raises the possibility of dysregulated follicular immune dynamics, in particular T-cell dynamics. To this end we developed imaging panels allowing for the *in-situ* detection, phenotyping, and quantitative analysis of different T- and B- cell subsets (**Supplementary Tables S2, S3**). First, assessment of geometrical characteristics of follicular areas (identified based on the density of CD19^hi/dim^ B cells) (**Figure 3A**) revealed that SLE follicles tend to be larger (p=0,08) and significantly less solid (more irregular boundaries) (p=0.008) compared to controls (**Figure 3B**) while a similar circularity was found between SLE and control LNS (**Supplementary Figure S2D**). T_FH_ cells represent the main follicular T cell population (40). Their indispensable role for the activation, maturation of B cells and the generation of high affinity antigen-specific antibodies are well established (42). The use of PD-1 as a T_FH_ cell biomarker (**Figure 3A**), showed comparable cell densities (cell counts normalized to mm^2^) of CD4^hi^PD1^hi^ T_FH_ cells between SLE and control follicles (**Figure 3C**). T_FH_ cells were further analyzed based on the expression of Ki67 and Bcl6 (**Figure 3D**). In line with previous reports (18, 43), only a subset of T_FH_ cells exhibited a proliferative capacity (**Figure 3E**, left panel). No significant differences were observed between control and SLE proliferating T_FH_ cells (**Figure 3E**, left panel). However, the cell density of T_FH_ cells expressing Bcl6, a master regulator of T_FH_ cells (43) was significantly downregulated(p<0,05) in SLE compared to control LNs (**Figure 3E**, right panel). Therefore, the significantly altered morphology of SLE follicles is associated with a dysregulated differentiation of T_FH_ cells characterized by the significantly reduced prevalence of Bcl6hi T_FH_ cells.

### Decreased levels of potential IL4-producing T_FH_ cell subsets and impaired *in situ* IL4-signaling in SLE follicles

**Figure 4:**
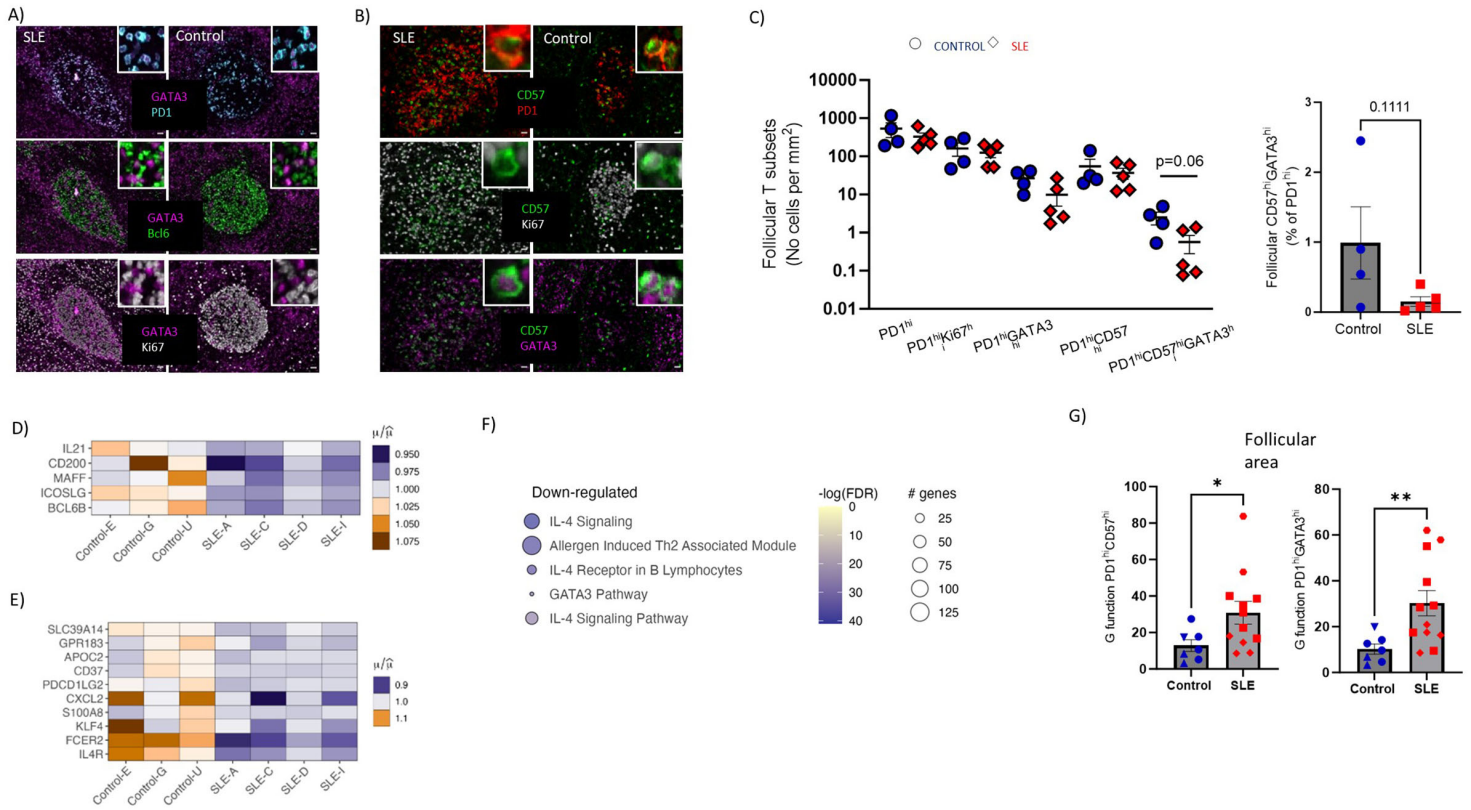
Underrepresentation of potential IL4-T_FH_ cell producers and impaired IL4-gene signature in SLE follicles. **(A)** Representative mIF images of GATA-3 (magenta), Bcl6 (green), Ki67 (grey) and PD-1 (cyan) from SLE and control LNs (scale bar:20mm). Zoomed areas are shown in white boxes. **(B)** Representative mIF images of GATA-3 (magenta), CD57 (green), Ki67 (grey) and PD-1 (red) from SLE and control LNs (scale bar:20mm). Zoomed areas are shown in white boxes. **(C)** Graph (left) demonstrating the follicular cell densities of PD1^hi^, PD1^hi^Ki67^hi^, PD1^hi^GATA3^hi^, PD1^hi^CD57^hi^ and PD1^hi^CD57^hi^GATA3^hi^ T_FH_ cells in SLE (N = 5) and control LNs (N = 4). Bar graph demonstrating the CD57^hi^GATA3^hi^ frequencies of total PD1^hi^ cells in SLE (N = 5) and control LNs (N = 4). Each dot/square represents one donor. The p values were calculated using the Mann–Whitney test. Data represent mean ± SEM. **(D)** Heatmap showing the relative mean expression of genes associated with T_FH_ differentiation. All genes displayed in **(D)** are significantly downregulated in SLE compared to control ROIs (P value < 0.05). **(E)** Heatmap showing the relative mean expression of IL4-stimulated genes. All genes displayed in **(E)** are significantly downregulated in SLE compared to control ROIs (P value < 0.05). **(F)** Reactome pathway analysis performed on differentially expressed genes of SLE and control follicular ROIs. The size of each node represents the number of genes involved in the corresponding Reactome term, while the color indicates the significance level of the enrichment (-logFDR). **(G)** Bar graphs showing the **(G)** function analysis for PD1^hi^GATA3^hi^ (right) and PD1^hi^CD57^hi^ (left) cells in individual follicles from control (N=7) and SLE (N=12) LNs. Each symbol represents a follicle. Different shapes represent different donors. The p values were calculated using the Mann–Whitney test. Data represent mean ± SEM. Only follicles harboring more than 20 cell events were analyzed. *P < 0.05; **P < 0.01.

The T_FH_ cell compartment is characterized by phenotypical and functional heterogeneity. A relatively high expression of GATA-3, a transcription factor associated with IL-4 production, has been described in human follicular areas by T_FH_ cells (19, 44, 45). Moreover, CD57 expression of T_FH_ cells has been associated with a unique position, molecular and functional profile (18, 46, 47). Furthermore, CD57^hi^ T_FH_ cells were found to be potent producers of IL-4 compared to CD57^lo^ T_FH_ cells, at least *in vitro (* 18). Therefore, we investigated the *in situ* phenotype of T_FH_ cells with respect to CD57 and GATA-3 expression (panel 3, **Supplementary Tables S2, S3**) (**Figures 4A, B**, **Supplementary Figure S3A**) by employing histo-cytometry analysis (**Supplementary Figure S3B**). In line with the data generated from our panel 1 (**Figures 3C, E**), we measured similar cell densities for PD1^hi^, PD1^hi^ Ki67^hi^ cells between SLE and control LNs (**Figure 4C**). However, a clear trend (p=0.06) for reduced PD1^hi^CD57^hi^GATA3^hi^ T_FH_ cell densities was revealed in SLE compared to control follicles (**Figure 4C**). A similar trend between control and SLE follicles was found when the relative frequencies of CD57^hi^GATA3^hi^ T_FH_ cells (% of PD1^hi^ cells) were plotted (**Figure 4C**, right panel).

To further investigate the perturbed differentiation of T_FH_ cells observed in SLE follicles, the gene expression of molecules that could mediate T_FH_ cell differentiation (*IL21*, *CD200*, *MAFF*, *ICOSLG*, *Bcl6b*) were analyzed using our spatial transcriptomic data. These genes, which can be expressed by more than one cell type, were downregulated in SLE compared to control follicles (**Figure 4D**). Taking into consideration that IL-4 is expressed mainly, if not exclusively, from T cells we reasoned that the reduced presence of potential IL4-producing T_FH_ cells would have an impact on IL4-related genes of SLE follicles. Indeed, SLE follicular areas exhibited a downregulated IL-4 signaling gene signature compared to controls, further supporting our imaging findings (**Figure 4E**). Furthermore, pathway analysis revealed that IL-4 related pathways were enriched in control compared to SLE follicles (**Figure 4F**). In addition to their capacity for secretion of critical cytokines (e.g. IL-21, IL-4), spatial positioning of T_FH_ cells is crucial for their optimal interaction with neighboring GC-B cells. To this end, the distribution profile (judged by the ‘G function’ parameter (48) of T_FH_ cell subsets was calculated. Follicles with at least 20 cells for each of the cell types were investigated, precluding thus the analysis of PD1^hi^CD57^hi^GATA3^hi^ T_FH_ cells. However, PD1^hi^CD57^hi^ and PD1^hi^GATA3^hi^ T_FH_ cells showed a significantly higher dispersed distribution in SLE compared to control follicles (**Figure 4G**) that could affect their interaction with B cells within the follicular areas. In conclusion, reduced cell densities of PD1^hi^CD57^hi^GATA3^hi^ cells in follicles could lead to impaired IL4-related responses, which may affect the development and the maturation of GC B cells.

### Bcl6^high^ GC B cells are significantly reduced in SLE LNs

**Figure 5:**
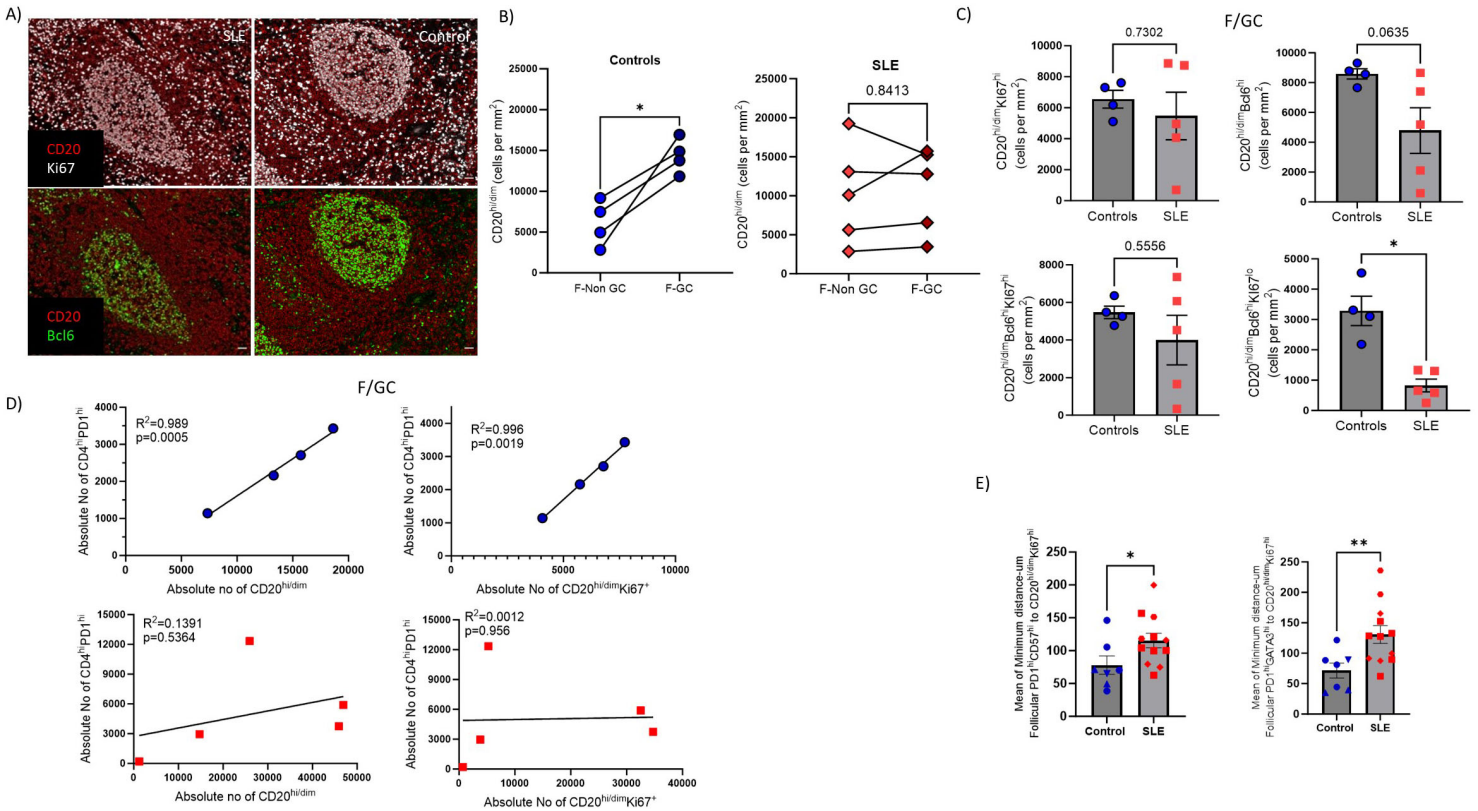
SLE F/GCs are characterized by reduced cell densities of CD20^hi/dim^Bcl6^hi^ B cells and by loss of association with T_FH_ cells. **(A)** Representative mIF images of CD20 (red), Bcl6 (green) and Ki67 (grey) staining patterns from SLE and control LNs (scale bar:20mm) **(B)** Dot plots demonstrating CD20^hi^ cell densities between GC and non-GC areas of control (left, N=4) and SLE (right, N=5) follicular areas. The p values were calculated using the Mann–Whitney test. **(C)** Bar graphs demonstrating the cell densities (cell counts normalized per mm^2^) of GC- CD20^hi/dim^Ki67^hi^ (upper left), CD20^hi/dim^Bcl6^hi^, (upper right), CD20^hi/dim^Bcl6^hi^Ki67^hi^ (lower left) and CD20^hi/dim^Bcl6^hi^Ki67^lo^ (lower right) B cells in SLE (N = 5) and control F/GCs (N = 4). Each dot/square represents one donor. The p values were calculated using the Mann–Whitney test. Data represent mean ± SEM. **(D)** Linear regression analysis between F/GC CD4^hi^PD1^hi^ with CD20^hi/dim^ (left) and between CD4^hi^PD1^hi^ and CD20^hi/dim^Ki67^hi^ (right) cell densities in controls (upper) and SLE (lower) follicles. Each symbol represents one donor. **(E)** Bar graphs showing the mean of minimum distance values (between PD1^hi^GATA3^hi^(right) or PD1^hi^CD57^hi^ (left) and CD20^hi/dim^ Ki67^hi^ in individual follicles from control and SLE LNs. Each symbol represents a follicle. Different shapes represent different donors. The p values were calculated using the Mann–Whitney test. Data represent mean ± SEM. Only follicles harboring more than 20 cell events were analyzed. *P < 0.05; **P < 0.01.

Then we focused our investigation on analyzing relevant B cell subsets (panel 1, **Supplementary Tables S2, S3**) in follicular Regions of Interest (ROIs) identified based on the expression pattern of CD20 and Ki67 (CD20^hi/dim^Ki67^lo^-follicular enriched in Mantle Zone, hereafter F-non-GC and CD20^hi/dim^Ki67^hi/dim^-follicular enriched in LZ/DL, hereafter F/GC) (**Figure 5A**, **Supplementary Figure S4A**). Contrary to SLE, a consistently higher number of CD20^hi/dim^ B cells in the F/GC compared to F/non-GC follicular area was monitored in control LNs (**Figure 5B**). Within the F/GC area, however, no significant differences of bulk CD20^hi/dim^ or CD20^hi/dim^Ki67^hi^ B cell density were observed between control and SLE LNs (**Figure 5C**, upper panel, **Supplementary Figure S4B**). Notably, a clear trend (p=0.0635) for lower cell density of CD20^hi/dim^Bcl6^hi^ B cells was found in SLE to control F/GCs (**Figure 5C**, upper panel). Proliferating CD20^hi/dim^ B cells expressing Bcl6 (CD20^hi/dim^Bcl6^hi^Ki67^hi^, dominating the Dark Zone) exhibited similar cell densities whereas their non-proliferating counterparts (CD20^hi/dim^Bcl6^hi^Ki67^lo^, mainly found in the Light Zone) were significantly decreased in SLE compared to control F/GCs (**Figure 5C**, lower panel).

Given the mutual regulation between T_FH_ and GC B cells (49), we asked whether the counts of these two immune cell types are correlated in our tissue cohort. A significant correlation between T_FH_ and GC-B cells was found only in control LNs further supporting our hypothesis of deregulated GC-responses in SLE (**Figure 5D**). As a surrogate of T/B cell proximity, presumably reflecting the possibility for their interaction too, we measured the minimum Euclidean distances between T_FH_ and CD20^hi/dim^Ki67^hi^ B cells (enriched in dark zone). The minimum number of cells required per F/GC for this analysis excluded PD1^hi^CD57^hi^GATA3^hi^ T_FH_ cells from this comparison. However, a significantly greater minimum distance between PD1^hi^CD57^hi^ or PD1^hi^GATA3^hi^ T_FH_ and CD20^hi/dim^Ki67^hi^ B cells was found in SLE compared to control F/GCs (**Figure 5E**) suggesting a lower possibility for T_FH_-B cell interaction in SLE. Therefore, SLE F/GCs are characterized by a concomitant dysregulated dynamics of both T_FH_ and B cells.

### Significant accumulation of autoreactive age-associated B cells in SLE

**Figure 6:**
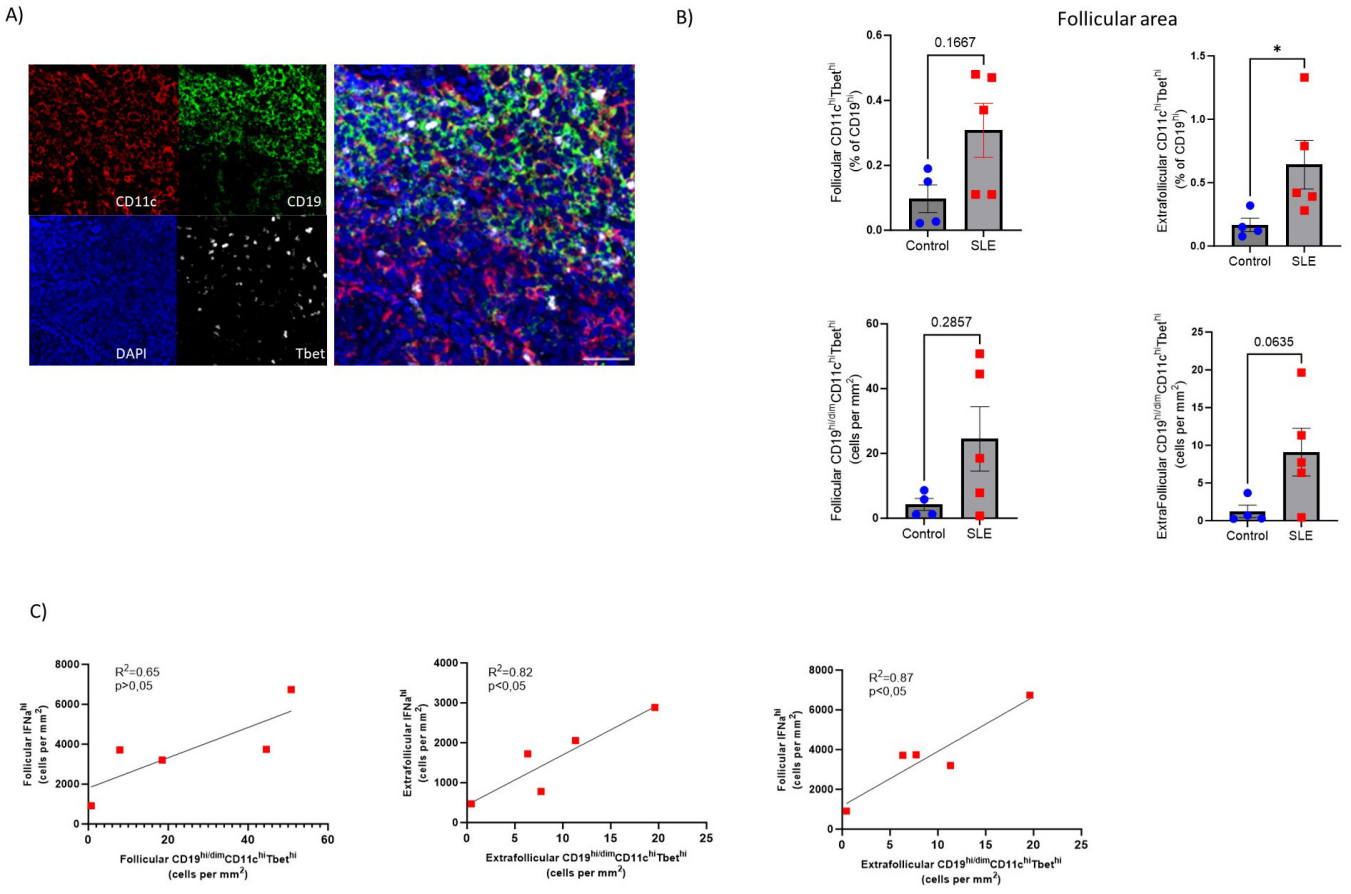
Accumulation of extrafollicular CD19^hi/dim^CD11c^hi^Tbet^hi^ B cells in SLE LNs **(A)** Representative mIF images of CD11c (red), CD19 (green), Tbet (grey) and DAPI (blue) from a SLE LN (40X, scale bar:30μM). **(B)** Bar graphs demonstrating the frequencies (upper, % CD11c^hi^Tbet^hi^ of CD19^hi/dim^ cells) and cell densities (lower) of follicular (left) and extrafollicular (right) CD19^hi/dim^CD11c^hi^Tbet^hi^ B cells in SLE (N=5) and control LNs (N=4). Each dot/square represents one donor. The p values were calculated using the Mann–Whitney test. Data represent mean ± SEM. **(C)** Linear regression analysis between follicular IFNα2^hi^ and follicular CD19^hi/dim^CD11c^hi^Tbet^hi^ (upper left), extrafollicular IFNα2^hi^ and extrafollicular CD19^hi/dim^CD11c^hi^Tbet^hi^ (upper right) and follicular IFNα2^hi^ with extrafollicular CD19^hi^CD11c^hi^Tbet^hi^ normalized cell counts (lower middle) of SLE LNs (N = 5). Each dot/square represents one donor. *P < 0.05.

Disturbed B cell differentiation/maturation can lead to the generation of pathogenic B cell subsets like Age-associated B cells (50). ABCs (CD19^hi/dim^CD11c^hi^Tbet^hi^) are crucial mediators of SLE autoreactive humoral responses (51). Our mIF assay (panel 4, **Supplementary Tables S2, S3** **) (** **Figure 6A**, **Supplementary Figure S4C**) showed increased prevalence (both as frequencies and cell density) of CD11c^hi/dim^Tbet^hi^ B cells in SLE follicular (CD19-dense areas) and especially extrafollicular areas (p<0,05), compared to control LNs, regardless the gender of SLE individuals (**Figure 6B**). Furthermore, we found a positive correlation between extrafollicular ABC and extrafollicular or follicular IFNα2^hi^ cell densities in SLE LNs (**Figure 6C**). Notably, the strongest positive correlation was observed between the follicular IFNα2^hi^ and extrafollicular ABC cell densities (**Figure 6C**). Therefore, the dominant follicular type I IFN signature and the increased generation of atypical B cell subsets in the extrafollicular area may represent molecular and cellular mechanisms contributing to the described dysregulated development of GC-B cell responses in SLE follicles.

### Significantly increased prevalence of IL21^hi^cells in SLE LNs

**Figure 7:**
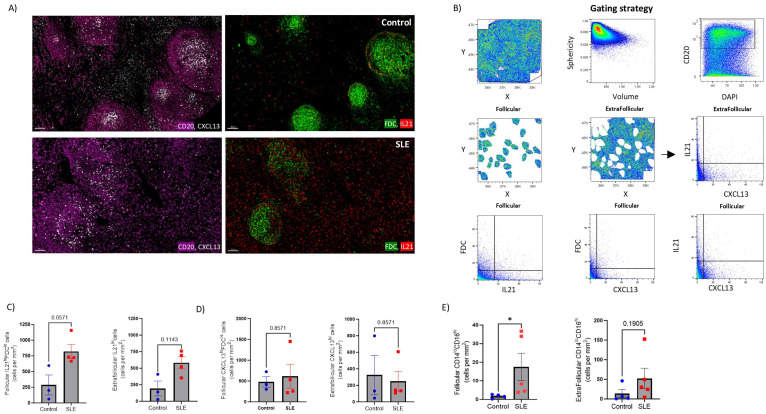
Significant accumulation of IL21^hi^ cells and non-classical monocytes in SLE follicles. **(A)** Representative mIF images of CD20 (magenta), FDC (green), CXCL-13 (grey) and IL-21 (red) from SLE and control LNs (scale bar:100 μM). **(B)** Histo-cytometry gating scheme used for the quantification of IL21^hi^ and CXCL13 ^hi^ FDC or non-FDC cell subsets. F and EF areas were manually identified based on the density of the CD20 signal gated back to the X, Y dot plot. Representative data from a control LN are plotted. **(C)** Bar graphs demonstrating the cell densities of follicular IL21^hi^FDC^lo^ (left) and extrafollicular IL21^hi^ (right) cells in SLE (N = 4) and control LNs (N = 3). Each dot/square represents a different donor. The p values were calculated using the Mann–Whitney test. Data represent mean ± SEM. **(D)** Bar graphs showing the cell densities of follicular CXCL13^hi^FDC^lo^ (left) and extrafollicular CXCL13^hi^ (right) cells in SLE (N = 4) and control LNs (N = 3). Each dot/square dot represents a different donor. The p values were calculated using the Mann–Whitney test. Data represent mean ± SEM. **(E)** Bar graphs demonstrating the cell densities of follicular (left) and extrafollicular (right) CD14^lo^CD16^hi^ cells in SLE (N = 5) and control LNs (N = 4). Each dot/square represents a different donor. The p values were calculated using the Mann–Whitney test. Data represent mean ± SEM.

Given the transcriptomic profile dominated by type I IFN in SLE follicles, we sought to analyze the expression of critical soluble mediators as well as innate immunity subsets in our tissue cohort. First, a mIF assay allowing for the analysis of CD20, FDC, IL-21 and CXCL13 (panel 5, **Supplementary Tables S2, S3**) was applied (**Figure 7A**). Our gating strategy allowed us to analyze IL2-1^hi^ and CXCL13^hi^ cells in both follicular and extrafollicular areas (**Figure 7B**). Contrary to FDC associated IL21 (**Supplementary Figure S5A**, left panel), follicular IL21^hi^FDC^lo^ positive cells were more abundant in SLE (p=0.057) compared to control follicles (**Figure 7C**, left panel). A similar but less evident profile (p=0.1143) was found for the extrafollicular IL21^hi^ cells (**Figure 7C**, right panel). No differences were found when the cell densities of CXCL13^hi^ cells were analyzed either in extrafollicular (**Figure 7D**) or follicular (**Figure 7D**, **Supplementary Figure S5** **A** right panel) areas of SLE and control LNs.

Follicular responses could also be directly or indirectly affected by innate immune cells and CD8^hi^ T cells (52–56). Analysis of bulk CD11c (panel 5, **Supplementary Tables S2, S3**, **Supplementary Figure S5B**) revealed a trend for higher CD11c^hi^ cells in the T cell zone (defined as CD4-dense extrafollicular areas) of control LNs (**Supplementary Figure S5C**). Analysis of CD14 and CD16 cell subsets (panel 4, **Supplementary Table S2**, **Supplementary Figures S5D, E**) showed similar cell densities for CD14^hi^ cells, in follicular and extrafollicular areas, among SLE and control LNs (**Supplementary Figure S5F**). Notably, non-classical CD14^lo^CD16^hi^ monocytes were increased in SLE follicles (**Figure 7E**, left panel). In the extrafollicular areas, CD14l°CD16^hi^ monocytes were also elevated in SLE compared to control LNs, however without reaching statistical significance (**Figure 7E**, right panel).

Next, the cell densities of bulk and effector CD8^hi^ T cells (panel 2, **Supplementary Tables S2, S3**) were analysed (**Supplementary Figure S5G**, upper panel). Similar cell densities of bulk and potential CTLs (GrzB^hi^Prf^hi^ CD8^hi^ T cells) were measured in the extrafollicular areas between SLE and control LNs (**Supplementary Figure S5G**, lower panel). However, a clear trend (p=0.057) for higher cell density of proliferating Ki67^hi^CD8^hi^ T cells was found in SLE compared to control LNs (**Supplementary Figure S5G**, lower panel). Collectively, these results indicate that SLE follicles exhibit alterations in IL-21-fuelled GC-reactivity and increased infiltration of inflammatory non-classical monocytes.

## Discussion

SLE is a complex autoimmune disease characterized by immune dysregulation, chronic inflammation, and multi-organ damage. Sustained type I IFN signaling, and autoantibody production create a vicious cycle that orchestrates SLE pathogenicity. With this in mind, we sought to investigate the immune cell landscape and dynamics of SLE LNs. In this study, we employed multiplex imaging and spatial transcriptomic to investigate for possible *in situ* cellular and molecular irregularities in follicles that could contribute to abnormal humoral SLE responses. We would like to emphasize that we utilized appropriate LNs (matched for anatomical location), characterized by follicular hyperplasia as a strict control for high GC reactivity. This approach allowed us to assess the cellular composition and the capacity of SLE LNs to be characterized by unique molecular signatures. It should be noted that we were unable to match our cohorts for gender due to the relative rarity of LN tissue samples from both groups. However, we did not observe any gender-related differences in the accumulation of ABCs and/or the type I IFN signature on SLE LNs, as suggested by some previous studies (57–61). We observed altered follicular morphology, characterized by increased area and boundary irregularities, in SLE compared to control LNs. Presumably, the ‘ectopic’ development of follicles found in SLE (62) contributes, at least in part, to this irregular formation.

To the best of our knowledge, this is the first study to provide spatial transcriptomic evidence for a profound type I IFN signature in SLE follicles, a molecular signature previously described in studies using blood as well as non-blood tissues (skin, kidney) and strongly correlated with SLE severity (63–65). In line with the transcriptomic data, our mIF analysis showed increased cell densities of IFNα2^hi^ cells, specifically in the follicles of SLE compared to control reactive LNs. The elevated and sustained IFN-response detected in SLE follicles could modulate adaptive and innate immune cell properties and be a crucial contributor for non-canonical GC and/or EF- responses as previously reported in SLE patients (50, 66) or SLE mouse models (7, 67). The concomitant expression of the follicular type I IFN fingerprint, reflected by the upregulation of type I interferon-related pathways, the downregulation of T_FH_ differentiation pathways and cytokine pathways (IL-1b) which are positive regulators of T_FH_ cell differentiation (68), urges for further investigation regarding the mechanistic link of these pathways. Interestingly, the detected plasma cell-related gene signature, dominating SLE follicles, agrees with previous peripheral blood transcriptomic studies (69). Therefore, the altered follicular morphology, the domination of type I interferon signaling and the downregulation of T_FH_ cell differentiation pathways represent tissue determinants that associate, at least, with a non-canonical development of GC B cells in SLE.

Given that GeoMx spatial transcriptomics platform does not provide single cell resolution, we used a computational deconvolution approach to delineate the contribution of different immune cell subsets to the detected type I IFN fingerprint of SLE follicles. We chose to deconvolute T, B, and dendritic cells to address whether follicular T or B cells are relatively more responsive to type I IFNs using dendritic cells as a positive control (70). As expected (70),, dendritic cells were more responsive to type I IFN than adaptive immune cells. Although it is expected that both follicular T and B cells are susceptible to IFN stimulation, our data suggest that follicular T cells, presumably T_FH_ cells, could be more affected by the immunomodulatory effect of type I IFNs compared to B cells. This is supported by the higher correlation of T cells on the expression of most of the randomly selected ISGs while B cell correlation was only found to be higher in the case of *IRF9* the role of which is less prominent compared to other well-established ISGs (71). We must emphasize that other cell subsets are also present in follicles and respond to type I IFNs, but we focused on these specific cell subsets for the scope of this investigation.

Despite the comparable cell densities of bulk PD1^hi^ T_FH_ cells among SLE and control LNs, a significant reduction of PD1^hi^Bcl6^hi^ T_FH_ cells was observed in SLE. T_FH_ cell differentiation is regulated by a complex network of transcription factors and signaling molecules, including members of the STAT family and several different cytokines including type I IFNs (16). However, the role of a particular molecule/pathway may differ with respect to the stage of a disease (e.g. acute vs chronic inflammation). Although type I IFNs can induce early differentiation of T_FH_ cells, i.e. through STAT1-mediated induction of Bcl6 (72, 73), in chronic infection they favor the development of Th1 instead of T_FH_ cell responses (74). Within the follicle, type I IFNs could act, at least in part, by downregulating Bcl6 (75, 76) which has been shown to bind ISGs loci and downregulate their expression in T_FH_ cells (77). Therefore, we hypothesize that the excessive type I IFN signaling could act as an underlying ‘orchestrator’ for the impaired development of Bcl6^hi^ T_FH_ responses in SLE follicles.

It should be noted that peripheral T cells (Tph), which are also characterized by elevated expression of PD1 and other T_FH_-related markers, have been reported to be of great importance in the pathogenesis of SLE (78–82). Although Tph exert their potent immunogenic properties mainly in inflamed tissues (82), they can also be present in the extrafollicular space of LNs where upon type I IFN stimulation can boost the LN immunoreactivity in a CXCL13-dependent manner as previously reported (81). This pathogenic T cell subset does not express CXCR5, which is required for intrafollicular migration (82) and its direct association with the F/GC Tfh cells that we immunophenotyped in our study is not well established. The multistep differentiation of T_FH_ cells can result in a heterogenous pool of cell subsets with distinct functional and positioning profile and presumably differential interaction and delivery of help to their B cell counterparts (15, 18, 47, 83). To gain insights into T_FH_ cell heterogeneity, we analyzed the expression of GATA-3, a transcription factor that shapes Th2 responses (45) and CD57, a senescence biomarker characteristic of highly differentiated T_FH_ cells (18), along with other T_FH_ cell biomarkers. SLE follicles exhibit decreased levels of highly differentiated GATA3^hi^ (PD1^hi^CD57^hi^GATA3^hi^) T_FH_ cells, further supporting our hypothesis of deregulated T_FH_ differentiation in SLE. The transition from PD1^hi^CD57^lo^ to PD1^hi^CD57^hi^ T_FH_ cells is associated with a reduced capacity for IL-21 production (18, 47) concomitant with increased secretion of IL-4, at least *in vitro (* 18). In line with this, we found a diminished IL4-related gene signature in SLE compared to control follicles. Type I IFN signaling, which can reverse human Th2 commitment by suppressing GATA-3 (84), could contribute, at least in part, to the described deregulated differentiation of T_FH_ cells towards a Th2-like phenotype. Additionally, the trend towards increased prevalence of proliferating and cytotoxic CD8^+^ cells might also boost a Th1-commitment of T_FH_ in an IFNγ-dependent fashion (48).

B cell maturation and differentiation into autoantibody-producing plasma cells can be T cell-dependent or T cell independent in SLE (11). Our findings suggest that B cell trafficking is altered in SLE F/GCs. The profile of CD20^hi/dim^ B cell prevalence between follicular non-GC (presumably MZ) and F/GC areas implies an altered B cell trafficking between follicular areas in SLE. Our transcriptomic analysis showed a relative downregulation of *CXCR5* (SLE vs Control: logFC=-0.2265, p-value=0.049) in SLE follicles that could presumably affect the trafficking of B cells (85). Whether the altered B cell trafficking is responsible, at least in part, for the altered SLE follicular morphology is not known and merits further investigation. On the other hand, the overall reduced cell density of CD20^hi/dim^Bcl6^hi^ B cells found in SLE was particularly evident for the LZ B cells (CD20^hi/dim^Bcl6^hi^Ki67^lo^), a profile also detected in the T_FH_ cell compartment. Our data indicate a generalized impairment in developing Bcl6^hi^ F/GC cell responses. Therefore, SLE follicular B cells could be affected both at differentiation and trafficking level. The mutual regulation that has been proposed between T_FH_ and GC B cells (15, 49) is likely impaired in SLE, because no association was found between T_FH_ and bulky or proliferating B cells, suggesting a ‘disconnection’, at least in part, between the two major GC immune cell types. We assume that the increased levels of peripheral T_FH_ cells reported in SLE (86) could reflect such non-canonical interaction with B cells in the follicles leading to pre-T_FH_ or T_FH_ cells egress from the lymph node.

Notably, we monitored upregulated levels of Age-associated B Cells (CD19^hi/dim^CD11c^hi^Tbet^hi^), a potent mediator of autoreactive humoral responses in SLE (57, 65, 87). More specifically, ABCs are tightly correlated with SLEDAI, renal involvement and autoantibody production (88). Extrafollicular CD20^hi/dim^ B cells are likely to bypass the canonical and tightly regulated GC-maturation triggered by cognate T-cell interactions and that could explain their autoreactive potential fueled by TLR-mediated signaling (89). Sustained type I IFN signaling can promote ABC generation by down-regulating IL4R on naïve B cells (50). Additionally, murine ABCs were reported to exhibit decreased levels of Bcl6 expression (57), in agreement with the reduced cell density of CD20^hi/dim^Bcl6^hi^ monitored in SLE compared to controls F/GCs. Interestingly, the presence of CD11c^+^Tbet^+^ B cells in SLE mouse models can induce an abnormal differentiation of T_FH_ cells creating thus a vicious feedback loop (90). In line with this, the reduced *in situ* IL-4-signaling, which is crucial for the GC B cell maturation (91–93), could fuel in SLE follicles the generation of ABCs by antagonizing the TLR-induced expression of Tbet in activated B cells (94). Moreover, T_ph_ cells, which are localized in extrafollicular areas and inflamed tissues, can possibly further promote ABC differentiation (80). Of note, a similar B-cell subset was up-regulated in lymph nodes from HIV infected individuals and associated with reduced capacity for broad neutralizing antibodies generation (95), further supporting the disruption of the normal GC responses by ABCs.

Analysis of innate immunity cell subsets revealed a higher cell density of ‘non-classical’ CD14^lo^CD16^hi^ monocytes especially in the follicles, that could be an additional source of inflammatory signals (96, 97). Our findings are in line with the increased circulating non-classical monocytes detected in SLE individuals (96). Among the local soluble mediators with major role in T_FH_ and B cells dynamics is the CXCL13 chemokine (98). We did not observe any difference between SLE and control LNs with respect to follicular and extrafollicular CXCL13. Although FDCs are the main source of CXCL13 (99), T_FH_ cells (100, 101), as well as circulating and tissue monocytes/macrophages (102–104) are also capable of secreting this chemoattractant. Our data suggest that this capacity is not significantly altered in SLE. Still, given the relatively reduced *CXCR5* mRNA found in SLE follicles by our spatial transcriptomic assay, the impact of an impaired function of the CXCL-13/CXCR-5 axis on T_FH_/B cell trafficking cannot be excluded. We found a clear trend for higher prevalence of IL21^hi^ cells, not associated with FDCs, in SLE, particularly in the follicular areas, in line with the increased frequency of IL21-producing circulating T_FH_ cells found in SLE (76). Interestingly, these cells can be expanded in a IFNα2-dependent manner (76). Whether trafficking of these cells back to LNs contribute to the increased IL21^hi^ cellular pool found in SLE needs further investigation. Regardless, the increase prevalence of IL21^hi^ cells could act as an additional positive regulator for the increased cell density of ABCs in SLE (105).

Despite the intriguing findings of our study, we should emphasize that the relatively low number of tissues examined limits the statistical power of our results. Future studies using larger cohorts of patients are needed to investigate the role of gender for the data provided in this study. However, obtaining relevant LNs from individuals prior to the initiation of a SLE treatment poses significant challenges since there is no medical indication for LN biopsies. Our data also cannot rule out the existence of a reverse mechanism where the inflammatory microenvironment leads to the generation of ABCs which in turn regulates T_FH_ IL-4 producing capacity in SLE (90). Nevertheless, the data point to a characteristic LN immune cell and transcriptomic landscape that may contribute to the SLE pathogenesis but also plays a role in the impaired i) immunity against viral infections (106) and ii) vaccine efficacy that has reported in SLE patients (25, 107–109).

In conclusion, our results suggest that *in situ* LN cellular and molecular irregularities characterized by i) sustained type I IFN signaling, ii) potent inflammatory signals (e.g., IL-21 and chemokines like CXCL9-CXCL11) iii) impaired generation of PD1^hi^CD57^hi^GATA3^hi^ T_FH_ cells and IL4-signaling and iv) accumulation of extrafollicular, potentially autoreactive, ABCs, could play an important role for the loose of tolerance and the generation of autoantibodies in SLE. In this context, the T_FH_/IL4-IL4R/ABC axis merits further investigation as it may provide druggable targets to alleviate SLE symptoms.

## Data Availability

The raw data supporting the conclusions of this article will be made available by the authors, without undue reservation. Presented data are accessible through https://doi.org/10.5281/zenodo.14615255 and GeoMx seq data, ENA ID PRJEB85597.
