# Supplementary material for: Cellular and molecular determinants mediating the dysregulated germinal center immune dynamics in systemic lupus erythematosus
Source: Front Immunol. 2025 Feb 13;16:1530327. doi: 10.3389/fimmu.2025.1530327 (PMC11894538; doi:10.3389/fimmu.2025.1530327)
Supplement: Supplementary file 1 [file DataSheet1.pdf]

Supplemental table 1. Demographic data of donors, clinical information and assays applied to each tissue/donor are listed.

ID	Location	Age	Gender	Autoimmune Manifestations	Used for
Control1(E)	Axillary	71	M	-	GeoMx, Panel1, Panel2, Panel3, Panel4, Panel5
Control2(F)	Cervical	39	M	-	Panel1, Panel2, Panel3, Panel4
Control3(G)	Inguinal	49	M	-	GeoMx, Panel1, Panel2, Panel3, Panel4, Panel5
Control4(U)	Inguinal	78	M	-	GeoMx, Panel1, Panel3, Panel4, Panel5
SLE1(A)	Inguinal	35	M	ANA (+), anti-SSA-52 (+), anti-SSA-60(+), C4(low), C3(low), isolated hemolysis, IgG=30.1 g/l	GeoMx, Panel1, Panel2, Panel3, Panel4, Panel5
SLE2(B)	Axillary	56	F	ANA (+), anti-nucleosome (borderline), SLE GN class V, IgG=10.7 g/l	Panel1, Panel2, Panel3, Panel4
SLE3(C)	Inguinal	21	M	ANA (+), anti-SSA-60 (+), anti-RNP (+), anti-Sm (+), anti-dsDNA (+), anti-nucleosome (+), C4(low), C3(low), leukopenia-anemia, IgG=47.5 g/l	GeoMx, Panel1, Panel2, Panel3, Panel4, Panel5
SLE4(D)	Inguinal	25	F	ANA (+), anti-SSA-52 (borderline), (+), anti-RNP (+), anti-Sm (+), anti- dsDNA (+), anti-nucleosome (+), C4(low), C3(low), leukopenia, IgG=23.1 g/l	GeoMx, Panel1, Panel2, Panel3, Panel4, Panel5

SLE5(I)	Cervical	21	F	ANA (+), anti-SSA-52 (+), anti-SSA-60(+), anti- dsDNA (+), anti-nucleosome (+), C4(low), C3(low), leukopenia-anemia, SLE GN class IV, IgG=14.78 g/l	GeoMx, Panel1, Panel3, Panel4, Panel5

Supplemental table 2. The antibody clones, catalogue numbers and fluorochrome used are shown.

**Epitope**	**Clone**	**Cat No**	**Fluorophore**
CD4	SP35	790-4423	Opal690
PD-1	NAT 105	ACI 3137	Opal620
Ki67	MIB-1	M7240	Opal780(1), Opal520(2)
CD20	L26	NCL-L-CD20-L26	Opal520
GATA-3	L50-823	760-4897	Opal570
Bcl-6	GI191E/A8	760-4241	Opal480
CD8	C8/144B	M710301-2	Opal780
PAX5	SP34	790-4420	Opal690
GRZb	GrB-7	MON7029C	Opal480
Perforin	5B10	Mob555	Opal570
CD57	NK-1	Mob 163	Opal480
CD14	EPR3653	114R-15	Opal520
CD16	2H7	CD16-L-CE	Opal570
CD11c	EP1347Y	ab52632	Opal780(4), Alexa555(5)
CD19	BT51E	NCL-L-CD19-163	Opal690

Tbet	MRQ-46	760-4598	Opal480
IFN-α2	Polyclonal	ab198914	Opal620
FDC	CNA.42	14-99-68-82	Alexa488
CXCL-13	Polyclonal	PA5-28827	Opal780
IL-21	Polyclonal	AHP1845	BV421

Supplemental table 3. The antibody panels and dilutions used are shown.

**Panel1**	**Panel2**	**Panel3**	**Panel4**	**Panel5**
Bcl-6 (Ready to use)	CD8 (1/100)	CD4 (Ready to use)	CD11c (1/200)	CD20 (1/400)
CD4 (Ready to use)	PD-1 (1/100)	PD-1 (1/100)	CD19 (1/200)	FDC (1/350)
PD-1 (1/100)	Perforin (1/10)	Ki67 (1/300)	Tbet (Ready to use)	IL-21 (1/80)
Ki67 (1/300)	GRZb (1/40)	CD20 (1/400)	IFN-α2 (1/200)	CXCL-13 (1/300)
CD20 (1/400)	PAX-5 (Ready to use)	GATA-3 (1/10)	CD14 (1/100)	CD11c (1/20)
GATA-3 (1/10)	Ki67 (1/300)	CD57 (1/200)	CD16 (1/100)	
DAPI	DAPI	DAPI	DAPI	SYTO45

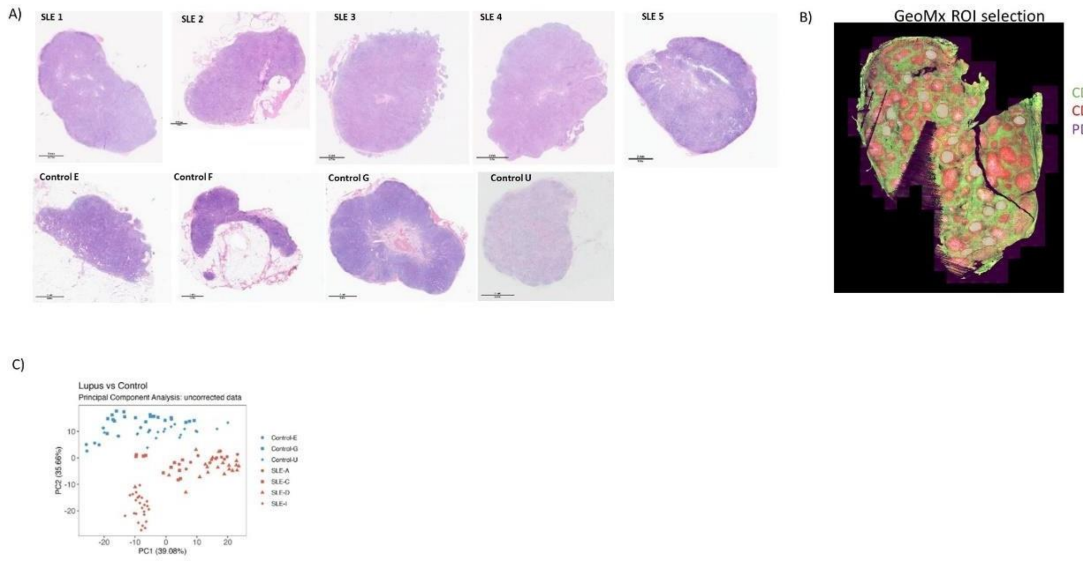

Supplemental Figure 1. (A) Hematoxylin and eosin (H&E) staining for all the LN tissues used in this study (scale bar: 1.5 mm). **(B)** Representative staining of one LN tissue using antibodies against CD3, CD20 and PD1 before GeoMx ROI selection.CD20-dense areas populated by CD3^{hi}PD1^{hi} cells were selected as ROIs (secondary mature follicles). **(C)** The PCA plot shows the distribution of spatial transcriptomic uncorrected data from 111 Regions of Interest (ROIs, follicles) collected from SLE (N=64, 4 donors) and control LNs (N=47, 3 donors). Each point represents an individual ROI, with the colour indicating the different cohort (Red=SLE, Blue=Control).

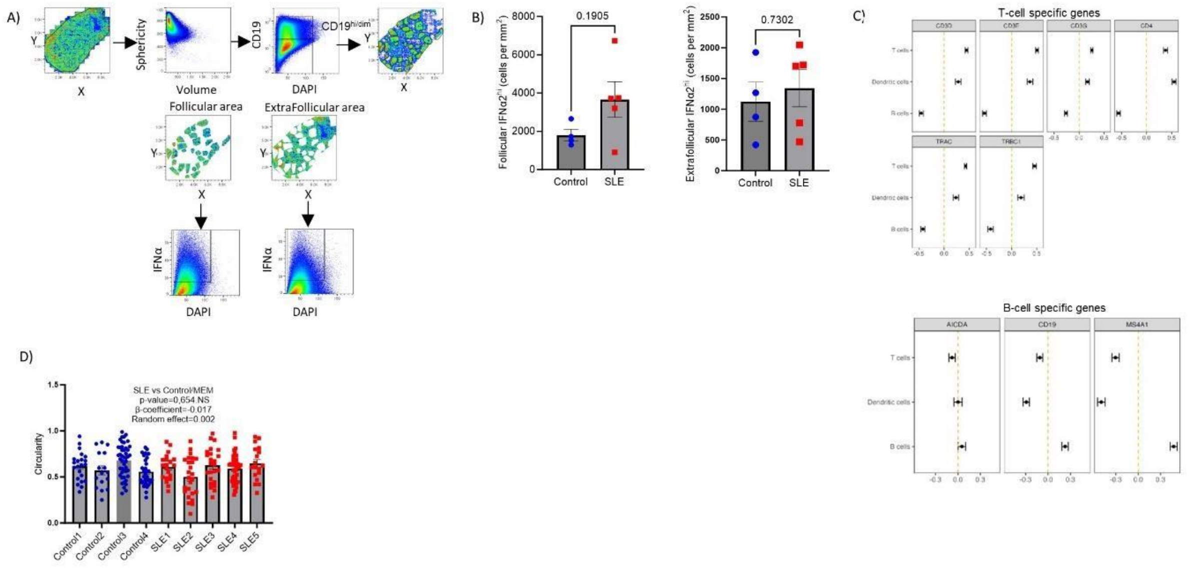

Supplemental Figure 2. (A) Histo-cytometry gating scheme used for the quantification of IFNα2^{hi} cells. F and EF areas were manually identified based on the density of the CD19 signal

and gated back to the X, Y dot plot. Representative data from a SLE LN are plotted. **(B)** Bar graphs demonstrating the cell densities of extrafollicular (right) and follicular (left) IFNα2^{hi} cells in SLE (N = 5) and control LNs (N = 4). Each dot/square represents one donor. The p values were calculated using the Mann–Whitney test. Data represent mean ± SEM. **(C)** Graphs representing the relative correlation of deconvoluted cell subsets proportion (T, B, dendritic cells) from all the ROIs with T (upper) and B (lower) cell related genes. **(D)** Bar graphs demonstrating the quantification of Circularity of follicles (identified as CD19$^{hi/dim}$ areas. Area and solidity of ROIs were calculated using FIJI. Each dot/square represents a different follicle. The p values were calculated using the mixed effects model (MEM). Data represent mean ± SEM.

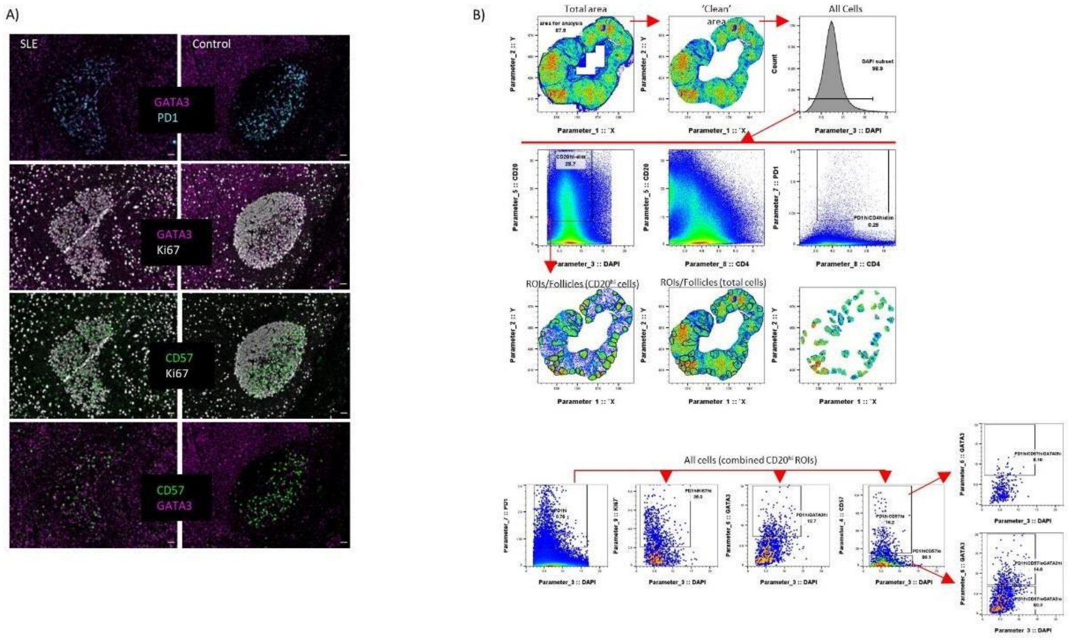

Supplemental Figure 3. (A) Representative mIF images of GATA3 (magenta), CD57 (green), Ki67 (grey) and PD1 (cyan) from SLE and control LNs (scale bar:20mm). **(B)** Histo-cytometry gating scheme used for the quantification of T_{FH} cell subsets. F and EF areas were manually identified based on the density of the CD20 signal and gated back to the X, Y dot plot. Representative data from a control LN are plotted.

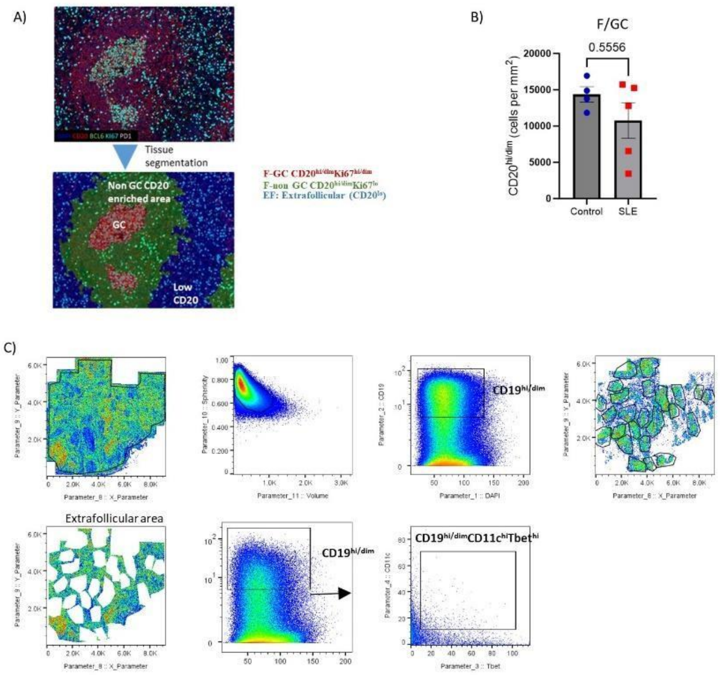

Supplemental Figure 4. (A) Representative example of tissue segmentation into different anatomical categories (F/GC-CD20$^{hi/dim}$Ki67$^{hi/dim}$- F/ non-GC-CD20$^{hi/dim}$Ki67^{lo}-EF CD20^{lo}) using the inForm image analysis software, version 2.4.8 (Akoya). **(B)** Bar graphs demonstrating the cell densities of F/GC- CD20$^{hi/dim}$ B cells in SLE (N = 5) and control LNs (N = 4). Each dot/square represents one donor. The p values were calculated using the Mann–Whitney test. Data represent mean ± SEM. **(C)** Histo-cytometry gating scheme used for the quantification of CD19$^{hi/dim}$CD11c^{hi}Tbet^{hi} cells. F and EF areas were manually identified based on the density of the CD19 signal and gated back to the X, Y dot plot. Representative data from a SLE LN are plotted.

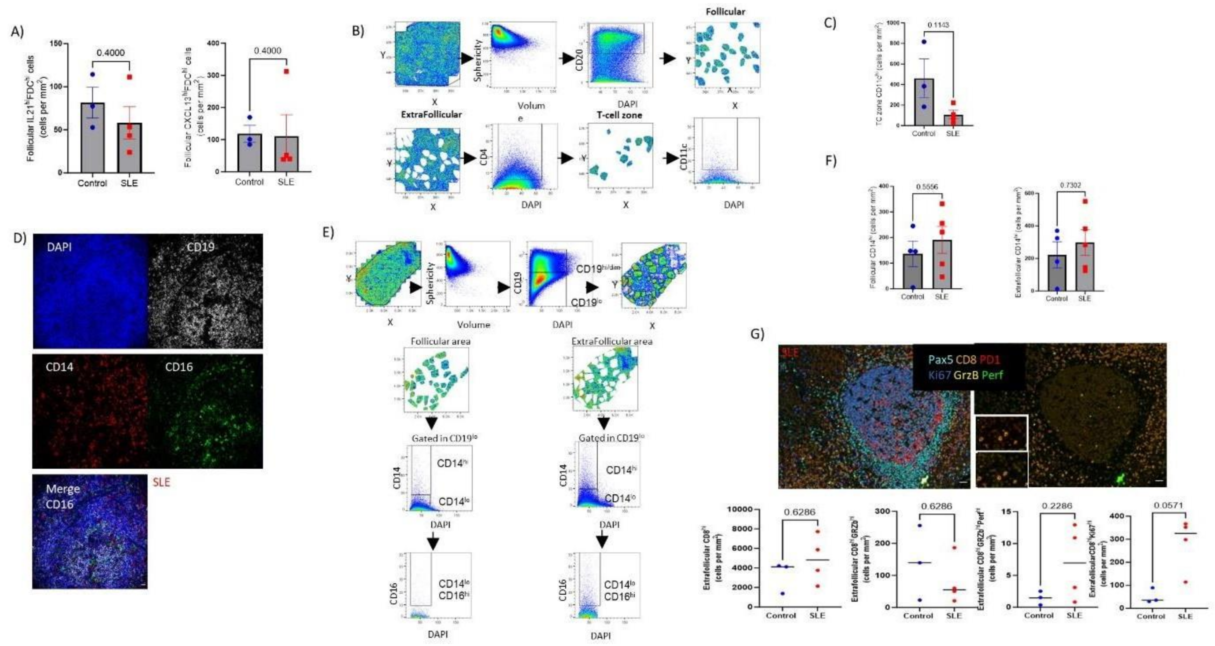

Supplemental Figure 5. (A) Bar graphs demonstrating the cell densities of follicular IL21^{hi}FDC^{hi} and CXCL13^{hi}FDC^{hi} cells in SLE (N = 4) and control LNs (N = 3). Each dot/square represents one donor. The p values were calculated using the Mann–Whitney test. Data represent mean ± SEM. **(B)** Histo-cytometry gating scheme used for the quantification of T-cell zone CD11c^{hi} cells. Real cells were gated based on their sphericity and volume. T-cell zone area was manually identified based on the density of the CD4 signal and gated back to the X, Y dot plot. Representative data from a control LN are plotted. **(C)** Bar graph demonstrating the cell densities of T-cell zone CD11c^{hi} cells in SLE (N = 4) and control LNs (N = 3). Each dot/square represents one donor. The p values were calculated using the Mann–Whitney test. Data represent mean ± SEM. **(D)** Representative mIF images of CD14 (red), CD16 (green) CD19 (grey) and DAPI (blue) from a SLE donor (40X, scale bar:30 μm). **(E)** Histo-cytometry gating scheme used for the quantification of CD14^{hi}, CD14^{lo}CD16^{hi} cell subsets. F and EF areas were manually identified based on the density of the CD19 signal and gated back to the X, Y dot plot. Representative data from a SLE LN are plotted. **(F)** Bar graphs demonstrating the cell densities of follicular (left) and extrafollicular (right) CD14^{hi} cells in SLE (N = 5) and control LNs (N = 4). Each dot/square represents one donor. The p values were calculated using the Mann–Whitney test. Data represent mean ± SEM **(G)** Representative examples of PD1 (red), CD8 (orange), GRZb (yellow), Perforin(green), Ki67 (blue) and Pax5 (cyan) staining patterns from a SLE LN (upper panel, scale bar:20mm). Bar graphs demonstrating the cell densities of extrafollicular CD8^{hi}, CD8^{hi} GRZb^{hi}, CD8^{hi} GRZb^{hi}Perf^{hi}, CD8^{hi} Ki67^{hi} cells in SLE (N = 4)

and control LNs (N = 3). Each dot/square represents one donor. The p values were calculated using the Mann–Whitney test. Data represent mean ± SEM.
